# Structural and compositional changes of whey protein and blueberry juice fermented using *Lactobacillus plantarum* or *Lactobacillus casei* during fermentation

**DOI:** 10.1039/d1ra04140a

**Published:** 2021-08-01

**Authors:** Wang Wen-qiong, Zhang Jie-long, Yu Qian, Zhou Ji-yang, Lu Mao-lin, Gu Rui-xia, Huang Yujun

**Affiliations:** College of Food Science and Engineering, Yangzhou University Yangzhou 225127 Jiangsu China wenqiong.happy@163.com zhoujiyang516@163.com lumlcn@yzu.edu.cn yjhuang@yzu.edu.cn rxgu@yzu.edu.cn Yzuzjl98@163.com yuqian20150811@163.com +86-13951434088 +86-19895320620 +86-18752540896 +86-514-87986305 +86-514-87978128 +86-17372713301 +86-17755853963; State Key Laboratory of Food Science and Technology, Jiangnan University Wuxi Jiangsu Province 214122 China; Weiwei Food & Beverage Co., Ltd Xuzhou 221114 Jiangsu China; Jiangsu Key Laboratory of Dairy Biotechnology and Safety Control, Yangzhou University Yangzhou Jiangsu China

## Abstract

This study aimed to improve the stability of the anthocyanins and phenolic acids of blueberry by forming hydrogen bonds or hydrophobic interactions with whey protein using lactic acid fermentation. The effects of the initial pH on the characteristics of the whey protein and blueberry juice system fermented using *Lactobacillus plantarum* and *Lactobacillus casei* were investigated. The color and total phenol and anthocyanin contents of the blueberry juice and whey protein system became stable after fermentation using *Lactobacillus plantarum* and *Lactobacillus casei*. Fluorescence measurements and Fourier transform infrared spectroscopy (FTIR) analysis reveal that the characteristics of whey protein and blueberry juice changed significantly after fermentation using *Lactobacillus plantarum* and *Lactobacillus casei* indicating the binding of anthocyanins or phenolic hydroxyl groups of blueberry to N–H, C–N and C=O groups of whey protein. The α-helix content of whey protein and blueberry fermented using *Lactobacillus plantarum* alone decreased by 18% and β-sheet content increased by approximately 27% compared to whey protein fermented using *Lactobacillus plantarum*.

## Abstract 2

A possible schematic diagram to show how the secondary structure changes which the whey protein and blueberry juice interaction after *Lactobacillus casei* or *Lactobacillus plantarum* fermentation was inferred from the structural analysis results.

## Graphical abstract

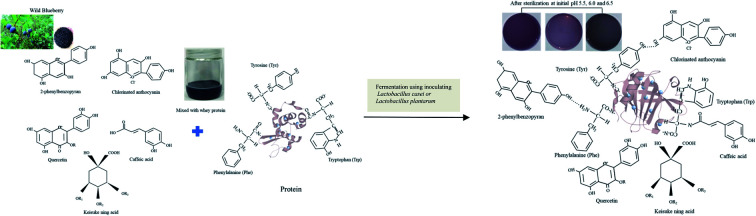

## 1. Introduction

Blueberry (*Vaccinium* spp., family Ericaceae), a very popular fruit in Northeast China, contains fiber, pectin, and phenolic compounds. Blueberry mainly contains anthocyanins, flavonols, and proanthocyanidins which have strong antioxidant properties.^1^ However, the physicochemical stability of anthocyanins including their color and structure is affected by many factors including pH, oxygen level, light, temperature, the presence of enzymes, and the surrounding components. Furthermore, the relatively poor oral and gastrointestinal absorption due to structural degradation, rapid metabolism and low permeability leads to a loss of functionality and bioavailability (Delfanian, *et al.*, 2020). Therefore, select appropriate to use this fruits as a source of natural colorants in processed foods is a great challenge in the blueberry beverage industry. There is already evidence that small intestinal epithelial cells can directly absorb anthocyanins, yet the transfer of anthocyanins from the oral cavity to the intestinal tract is quite different because of their instability in the gastrointestinal environment.^2^ Today, people are more inclined to purchase healthier dietary foods without compromising their quality. Distinct components in foods can interact with each other by forming of conjugates, which may improve the functionality and nutritional value of products.^3^ The interaction between different components in food and their new formed during production are more important than its natural state. The stability of anthocyanins can be enhanced by forming non-covalent complexes between biopolymers and phenolic compounds. The interaction between anthocyanins and pectin *via* the formation of hydrogen bonds or hydrophobic interactions could improve the stability of anthocyanins and the polysaccharide.^4^ Protein–polysaccharide complexes have shown notable functional properties, such as decreasing aggregation and precipitation of proteins, improving the stability of proteins during heat treatment, and modifying of the viscosity of blended systems.^5^ Whey proteins are soluble under acidic conditions, and are usually used to produce protein-grafted phenol or polysaccharide complexes. The surface pressure and dilatational properties of the green tea polyphenols-β-lactoglobulin nanocomplexes were found to be lower than those of pure β-lactoglobulin.^6^ Many studies have attempted to encapsulate of phenolics using delivery systems to improve their half-stability and functionality in fortified foods. Lactic acid bacteria (LAB) fermentation can increase the content of free absorbable phenolic acids because of the release of bound phenolic acids from the fiber. Further, the antioxidant activity can be improved by increasing the polyphenol content, which results from microbial hydrolase activity. The fermentation of mulberry pomace using *Lactobacillus plantarum* had positive effects on the phenolic compounds contents and the antioxidant activity, which might be associated with the production of new phenolic acids and the increase in the aglycone and phenolic acid concentrations.^7^ A good interaction was observed between *Lactobacillus casei* and these phenolic compounds. The addition of phenolic compounds and *Lactobacillus casei* could increase the functional properties of ice cream without causing a negative effect organoleptically.^8^*Lactobacillus casei* is a probiotic microorganism, intentionally introduced into fruits and vegetable juices because of its high activity and survivability. The carrot-based *Lactobacillus casei* fermented beverage can be served as a ready-to-drink product for 6 weeks under refrigerated storage, meeting the standards (10^8^–10^10^ CFU mL^−1^) of a functional drink. These probiotic microorganisms can also play a protective role against pathogens in the product itself during storage by competing with pathogens for nutrients, producing organic acids and bacteriocins.^9^

In this study, *Lactobacillus casei* and *Lactobacillus plantarum* were used to ferment whey protein and blueberry at different initial pH to ensure the stability of blueberry anthocyanins under acidic conditions. It has been reported that whey proteins have the ability to protect anthocyanins in the stomach according to *in vitro* digestibility tests.^10^ Therefore, we attempted to use microbial acid production or the interaction with substances to make the whey protein interact with the bioactive ingredients from blueberry during fermentation, making the system stable. Structure change and active ingredient analysis were used to determine the chemical bond formation between whey protein and blueberry active ingredient during fermentation.

## 2. Materials and methods

### 2.1 Materials

Whey protein was purchased from Fonterra (Auckland, New Zealand); it contained 80% protein, 4.5% moisture, 5% fat, 5% lactose, and 4.5% ash. Blueberry samples were collected during the 2019 harvest period in Daxinganling, China (51°55′ N, 124°34′ E). *Lactobacillus casei* and *Lactobacillus plantarum* were supplied by Shanghai HOWYOU Food Science Co., Ltd.

### 2.2 Preparation of the blueberry and whey isolate protein fermentation beverage

All samples were stored at −20 °C during the analysis. The inoculum was prepared using reconstituted (12% w/v) skim milk powder. Blueberry juice preparation was also prepared. Nine beverages were formulated with no added sugar, and containing 100 mL blueberry juice and 6% (w/v) whey protein. The mixtures were heated to 95 °C for 50 s. After cooling the mixtures to 25 °C, the culture inoculum of *Lactobacillus casei* and *Lactobacillus plantarum* were added at 0.2% (v/v). The fermentation temperature was 37 °C. The samples were refrigerated until the consumer testing.

### 2.3 Color analysis

To study the changes in the beverage color caused by the changes that occur during fermentation, the color of each sample was measured using a Minolta Chroma Meter CR-400 colorimeter (Minolta Ltd, Milton Keynes, UK). The measurement results are in the form of L*, a* and b*. a* reflects the trend of redness to greenness, b* reflects the trend of yellowness to blueness. Each sample was placed in a cuvette and then measured using machine. Each sample was measured thrice.^11^ A colorimeter was used to record the color change of the beverage during fermentation and to calculate the ΔE*, ΔL*, Δa*, and Δb* values. Here, ΔE* = (ΔL*^2^ + Δa*^2^ + Δb*^2^)^1/2^, where ΔL*, Δa*, and Δb* are the differences between the color coordinates before and after fermentation. Images of the overall appearance of the samples were recorded with a digital camera at the same time.^12^

### 2.4 Determination of the total phenolic acids

The total phenolic content of the samples were measured by Foline Ciocalteau method. A volume of 0.15 mL of the sample solution, 0.15 mL of Folin-phenol reagent and 1.5 mL of a 75 g L^−1^ Na_2_CO_3_ solution were added to the centrifuge tube. After mixing evenly and resting for 5 min, 3.2 mL of deionized water was added to a final 5 mL. The tube was placed in a 50 °C electric constant temperature water bath in the dark for 5 minutes. The absorbance was measured at 750 nm using a UV-spectrophotometer (Model 722, Jingke Meter Company, Shanghai, China). The results are expressed as milligrams of gallic acid equivalents per gram of fresh weight using a standard curve with gallic acid concentrations at 1.25–25 mg L^−1^.^13^

### 2.5 Determination of total anthocyanin content

The typical pH-differential method was used to assess the differences in total anthocyanin content (TAC). Briefly, 0.025 mol L^−1^ KCl buffer (adjust pH to 1.0 using HCl) and 0.4 mol L^−1^ CH_3_COONa buffer (adjust pH to 4.5 using HCl) were prepared in advance. Then, mix 1 mL of sample and 9 mL of each of the above two buffers respectively, equilibrated for 15–30 min, and the absorbance values were measured at 520 nm and 700 nm, respectively. The calculation formulas were as follows:

1 Δ*A* = (*A*_520_ − *A*_700_)pH 1.0 − (*A*_520_ − *A*_700_)pH 4.5

2 TAC = (Δ*A* × MW × DF × 100)/(*ε* × *l*)

where Δ*A* is the absorbance difference, DF is the dilution factor, MW is the molecular weight of cyanidin-3-glucoside (449.20 g mol^−1^), *ε* is the molar absorptivity of cyanidin-3-*O*-glucoside (26 900 L mol^−1^ cm^−1^), and *l* is the path length of 1 cm. Measurements were performed in triplicate.^1^

### 2.6 Fluorescence spectra

Fluorescence measurements were performed using a RF-5301 spectrofluorometer (Shimadzu Corp., Tokyo, Japan). All samples were mixed with phosphate-buffered saline (pH 7.4) at room temperature. Each sample was diluted to a concentration of 0.005 g mL^−1^ (w/v) using the same buffer. The fluorescence spectra were recorded at an excitation wavelength of 290 nm and emission wavelength from 350 to 380 nm. Spectra were further analyzed using Origin 8.0 program (OriginLab, Northampton, MA, USA).^14,15^

### 2.7 FTIR

The samples were tran into lyophilized powder beforehand and then measured using a Varian Cary 610/670 FTIR spectrometer in ATR mode. The parameters were set as follows: the mode of the Ever Glo infrared source was Turbo and 36 scans were performed with a selected resolution of 8 cm^−1^.

### 2.8 Circular dichroism (CD) spectroscopy

The secondary structure of fermented whey protein and blueberry was determined by circular dichroism polarimeter (Jasco J-810, Jasco Corporation Japan) at 25 °C. The spectral resolution was 0.5 nm, ranging 190–250 nm, and a scanning speed of 100 nm min^−1^ and the sensitivity of 20 mdeg. The concentration of the sample was diluted to a concentration of 0.2 mg mL^−1^. A protein secondary structure was estimated according to the method of the DICHROWEB program CONTINLL.^16,17^

### 2.9 Scanning electron microscopy (SEM)

The samples were dehydrated using a freezer dryer (LGJ50; SiHuan Ltd, Beijing, China). The microstructure of the fermented samples was examined by SEM (GeminiSEM300, Carl Zeiss Ltd, England).

## 3. Results and discussion

### 3.1 The pH changes of whey protein and blueberry juice fermentation systems

**Fig. 1:**
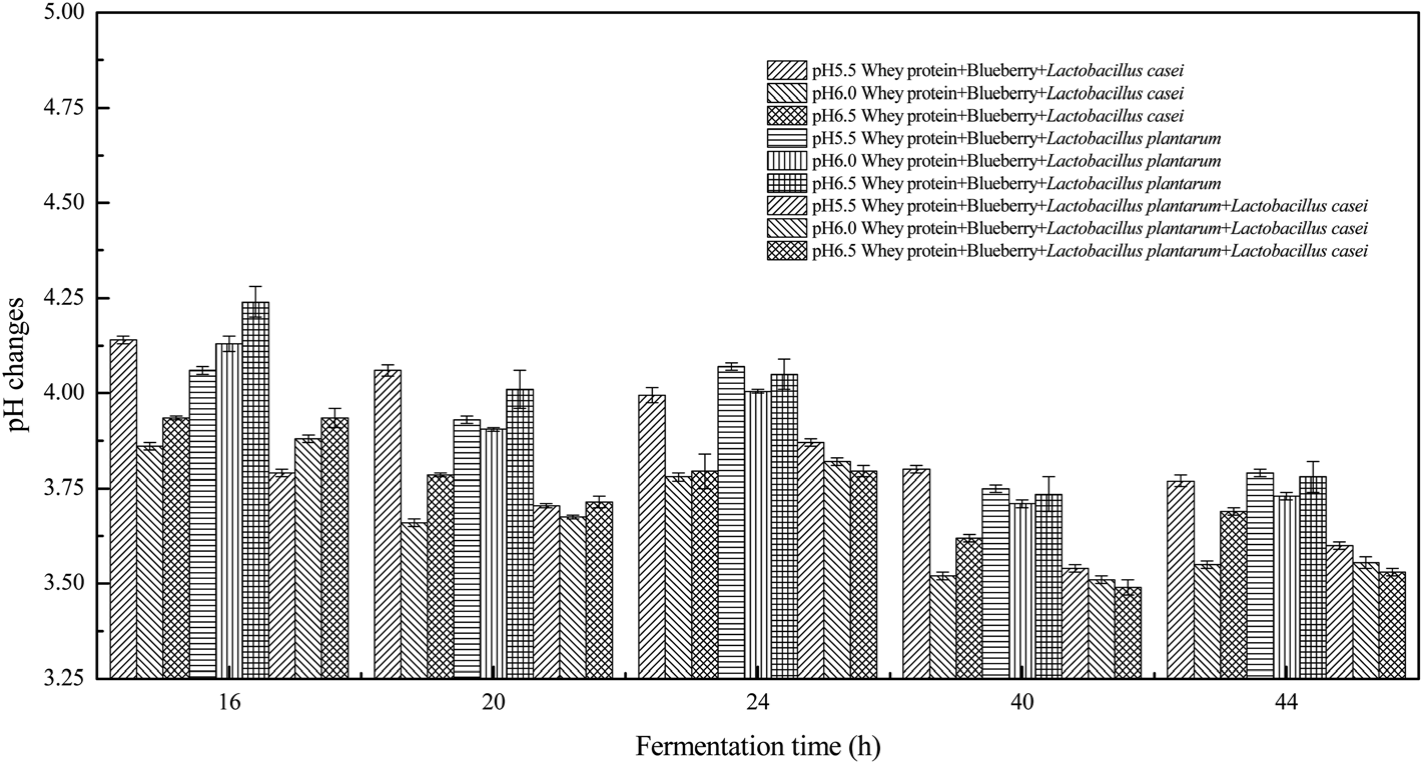
The pH changes of whey protein and blueberry juice fermented using *Lactobacillus plantarum*, *Lactobacillus casei* or their combination systems with initial pH 5.5, pH 6.0 and pH 6.5 conditions at the fermentation time of 16 h, 20 h, 24 h, 40 h and 44 h.

The initial medium pH may affect cell membrane function, cell morphology and structure, the solubility of salts, the ionic state of substrates, the uptake of various nutrients, and product biosynthesis. In general, cells can only grow within a certain pH range, and metabolite formation is often affected by the pH value.^18^ Therefore, the effect of initial pH on whey protein and blueberry fermented systems was investigated. As shown in Fig. 1, the pH decreased as the fermentation time increased and was stable for 40 h of fermentation. The initial high fermentation pH resulted in a relatively higher fermentation terminal pH. Different initial fermentation pH values resulted in various terminal pH values, which also favored different microbes. The combined *Lactobacillus plantarum* and *Lactobacillus casei* fermented whey protein and blueberry mixture had a lower pH during fermentation. When the blueberry was added to whey protein, the pH sharply decreased during fermentation using *Lactobacillus casei* with an initial pH of 6.0. The same trend was for pH 6.5. However, the pH of whey protein and blueberry fermented using *Lactobacillus casei* decreased slowly at initial pH of 5.5 and was higher than that observed at an initial pH 6.0 or and 6.5. The pH of whey protein fermented using *Lactobacillus plantarum* at an initial pH of 6.0 was higher than that of the blueberry addition sample, indicating that blueberry promoted *Lactobacillus plantarum* acid production.

### 3.2 Measurement of the color change in fermentation system

Color is one of the most important beverage consumption indicators. pH was directly related to the level of pseudobase (colorless) and inversely related to the cation in the presence of oxygen. In addition, pH and temperature have been reported to be two critical factors that influence color degradation in lowbush blueberry products.^19^Table 1 shows the changes in the color parameters of whey protein and blueberry juice fermentation systems with initial pH values of 5.5, 6.0 and 6.5. W + C: whey protein + blueberry + *Lactobacillus casei*; W + P: whey protein + blueberry + *Lactobacillus plantarum*; and W + C + P: whey protein + blueberry + *Lactobacillus casei* + *Lactobacillus plantarum*. Table 1 also shows the visual color changes of the beverage. ΔL* represents the brightness of the beverage. The larger the value, the brighter the beverage was. According to Table 1 among different fermentation systems, during the entire fermentation process, the change in brightness at pH 6.5 is the highest and that at pH 5.5 is the lowest, which is not markedly affected by the strain used. Δa* indicates that the red and green color of the coloring substances in the beverage are biased. If Δa* is positive, the beverage is reddish; if Δa* is negative, the beverage is greenish. All fermentation systems gradually turned red. The fermentation system with an initial pH of 6.0 was faster than those with initial pH values of 5.5 and pH 6.5. The fermentation system with *Lactobacillus plantarum* was faster than that with *Lactobacillus casei*. The Δb* value indicates yellow-blue bias of the coloring substances in the beverage. When the Δb* value is positive, the beverage was to yellowish. A negative Δb* indicates the beverage was biased to blue. The fermentation system with initial pH of 5.5 gradually turned yellow. The fermentation system with initial pH of 6.0 was initially blue and then turned yellow after 24 h of fermentation. The fermentation system with an initial pH of 6.5 turned blue. For initial pH values of 5.5 and 6.0, the color became stable until fermentation for 40 h. As shown in Table 1, the color of beverage before fermentation was darker than that of the fermented samples. Even the beverage at an initial pH of 6.5 turned to black after sterilization. However, the color turned purple-red after fermentation; this was due to the acid conditions established after fermentation for 40–44 h, when the pH maintained at 3.5–4.0, as shown in Fig. 1. Anthocyanins can exist as stable cations under acidic conditions. Anthocyanins can become unstable chalcones as the pH gradually increases. Therefore, the color stability of anthocyanins can be improved *via* microbial fermentation.

**Table 1:** Color parameters for whey protein and blueberry juice fermentation systems with initial pH 5.5, pH 6.0 and pH 6.5 conditions during fermentation time. W + C: whey protein + blueberry + *Lactobacillus casei*; W + P: whey protein + blueberry + *Lactobacillus plantarum*; W + C + P: whey protein + blueberry + *Lactobacillus casei* + *Lactobacillus plantarum*. The images for whey protein and blueberry juice fermentation systems with initial pH 5.5, pH 6.0 and pH 6.5 conditions before (including before and sterilization) and after fermentation. (a) Whey protein + blueberry + *Lactobacillus casei*; (b) whey protein + blueberry + *Lactobacillus plantarum*; (c) whey protein + blueberry + *Lactobacillus casei* + *Lactobacillus plantarum*

Samples	W + C	W + P	W + C + P	W + C	W + P	W + C + P	W + C	W + P	W + C + P	W + C	W + P	W + C + P	W + C	W + P	W + C + P
pH 5.5	16 h			20 h			24 h			40 h			44 h		
ΔL*	12.38 ± 0.044^a^	10.81 ± 0.058^c^	11.21 ± 0.03^b^	10.86 ± 0.052^c^	10.27 ± 0.061^e^	10.61 ± 0.05^d^	10.70 ± 0.06^d^	9.55 ± 0.017^g^	9.98 ± 0.022^f^	8.46 ± 0.049^h^	7.49 ± 0.035^ij^	7.66 ± 0.029^i^	7.61 ± 0.064^i^	6.65 ± 0.023^k^	7.21 ± 0.043^j^
Δa*	11.43 ± 0.046^j^	11.92 ± 0.042^h^	11.65 ± 0.020^i^	12.65 ± 0.023^f^	12.77 ± 0.058^f^	12.40 ± 0.06^g^	13.62 ± 0.05^d^	13.72 ± 0.02^d^	13.06 ± 0.035^e^	15.54 ± 0.06^a^	15.41 ± 0.041^b^	14.97 ± 0.043^c^	12.74 ± 2.75^a^	15.40 ± 0.015^b^	15.33 ± 0.04^b^
Δb*	4.62 ± 0.009^n^	5.60 ± 0.013^i^	5.57 ± 0.006^j^	5.25 ± 0.003^m^	5.77 ± 0.007^h^	5.76 ± 0.009^h^	5.39 ± 0.007^k^	6.00 ± 0.012^g^	6.01 ± 0.003^g^	6.53 ± 0.003^f^	6.01 ± 0.003^d^	6.64 ± 0.006^c^	6.46 ± 0.007^e^	6.88 ± 0.006^a^	6.77 ± 0.013^b^
ΔE*	17.47 ± 0.006^h^	17.04 ± 0.003^m^	17.10 ± 0.01^k^	17.47 ± 0.034^h^	17.37 ± 0.010^i^	17.30 ± 0.014^j^	18.13 ± 0.01^e^	17.76 ± 0.01^g^	17.49 ± 0.015^h^	18.72 ± 0.03^a^	18.33 ± 0.020^c^	18.07 ± 0.026^f^	18.39 ± 0.01^b^	18.13 ± 0.003^e^	18.19 ± 0.02^d^
pH 6.0	16 h			20 h			24 h			40 h			44 h		
ΔL*	15.86 ± 0.023^c^	16.40 ± 0.023^a^	15.45 ± 0.012^d^	15.87 ± 0.022^c^	15.91 ± 0.038^c^	15.29 ± 0.035^e^	16.13 ± 0.019^b^	14.47 ± 0.006^g^	14.41 ± 0.033^g^	14.59 ± 0.017^f^	13.02 ± 0.023^i^	12.65 ± 0.009^j^	13.59 ± 0.027^h^	12.70 ± 0.009^j^	12.21 ± 0.02^k^
Δa*	13.45 ± 0.023^m^	12.78 ± 0.030^o^	13.87 ± 0.023^k^	13.37 ± 0.021^n^	14.24 ± 0.037^i^	14.44 ± 0.026^h^	14.05 ± 0.020^j^	14.53 ± 0.02^g^	14.90 ± 0.007^f^	15.60 ± 0.03^e^	15.85 ± 0.025^d^	16.28 ± 0.010^b^	14.64 ± 0.28^e^	14.74 ± 0.233^c^	15.09 ± 0.09^a^
Δb*	−0.22 ± 0.003^j^	−0.20 ± 0.003^j^	−0.16 ± 0.007^i^	0.12 ± 0.010^g^	−0.21 ± 0.009^j^	−0.09 ± 0.012^h^	0.17 ± 0.003^fg^	0.19 ± 0.000^f^	0.19 ± 0.012^f^	0.48 ± 0.006^e^	0.48 ± 0.009^e^	0.64 ± 0.007^d^	0.90 ± 0.009^a^	0.70 ± 0.003^c^	0.74 ± 0.009^b^
ΔE*	20.81 ± 0.003^c^	20.79 ± 0.007^c^	20.77 ± 0.00^c^	20.75 ± 0.010^cd^	21.36 ± 0.006^ab^	21.04 ± 0.00^b^	21.40 ± 0.00^a^	20.51 ± 0.01^g^	20.74 ± 0.01^d^	21.38 ± 0.01^a^	20.52 ± 0.016^g^	20.63 ± 0.012^e^	20.67 ± 0.00^d^	20.57 ± 0.020^f^	20.43 ± 0.02^h^
pH 6.5	16 h			20 h			24 h			40 h			44 h		
ΔL*	18.48 ± 0.658^ab^	18.54 ± 0.035^ab^	18.99 ± 0.023^a^	19.18 ± 0.055^a^	18.09 ± 0.041^b^	18.71 ± 0.04^a^	19.07 ± 0.03^a^	17.70 ± 0.01^b^	19.03 ± 0.04^a^	17.99 ± 0.01^b^	16.71 ± 0.007^c^	17.36 ± 0.020^bc^	17.01 ± 0.015^c^	12.83 ± 3.313^cd^	17.32 ± 0.015^bc^
Δa*	12.79 ± 0.046^k^	13.91 ± 0.032^h^	13.25 ± 0.017^j^	13.24 ± 0.055^j^	14.40 ± 0.033^f^	13.85 ± 0.050^h^	13.62 ± 0.038^i^	14.70 ± 0.028^e^	14.03 ± 0.026^g^	15.02 ± 0.012^d^	15.72 ± 0.000^b^	14.96 ± 0.032^d^	13.75 ± 0.657^c^	13.49 ± 0.220^a^	13.23 ± 0.204^c^
Δb*	−1.94 ± 0.015^e^	−2.28 ± 0.007^j^	−2.15 ± 0.000^i^	−1.94 ± 0.013^e^	−2.12 ± 0.006^h^	−2.16 ± 0.009^i^	−1.81 ± 0.012^d^	−2.02 ± 0.012^f^	−2.06 ± 0.012^g^	−1.92 ± 0.009^e^	−1.81 ± 0.003^d^	−1.69 ± 0.007^c^	−1.42 ± 0.003^a^	−1.68 ± 0.009^c^	0.65 ± 2.243^b^
ΔE*	22.55 ± 0.532^c^	23.28 ± 0.010^b^	23.25 ± 0.010^b^	23.38 ± 0.017^b^	23.22 ± 0.012^b^	23.37 ± 0.009^b^	23.50 ± 0.010^b^	23.09 ± 0.005^b^	23.73 ± 0.018^a^	23.51 ± 0.005^b^	23.00 ± 0.005^bc^	22.97 ± 0.005^bc^	22.88 ± 0.004^bc^	22.70 ± 0.017^bc^	23.25 ± 0.168^b^
The images of the system color	Before sterilization at different initial pH			After sterilization at different initial pH			After fermentation at initial pH 5.5			After fermentation at initial pH 6.0			After fermentation at initial pH 6.5		
	pH 5.5	pH 6.0	pH 6.5	pH 5.5	pH 6.0	pH 6.5									
	a1	b1	c1	a2	b2	c2	a3	b3	c3	a4	b4	c4	a5	b5	c5
	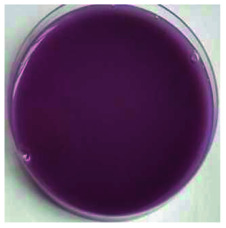	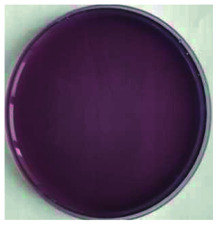	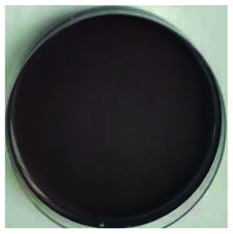	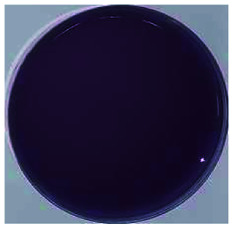	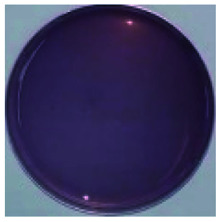	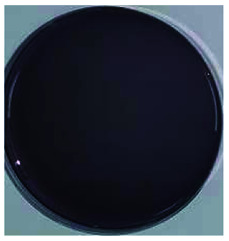	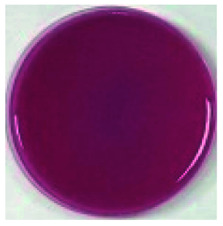	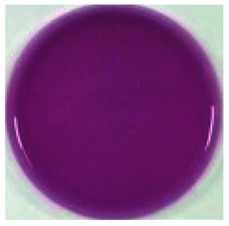	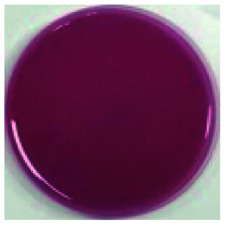	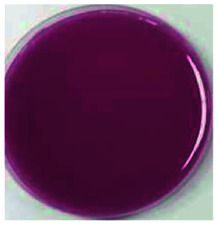	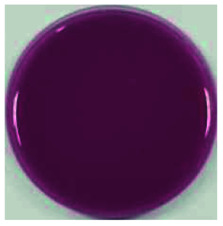	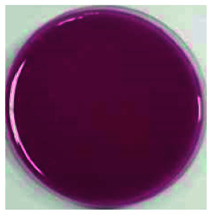	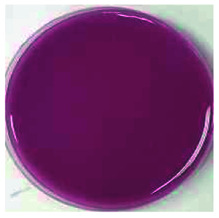	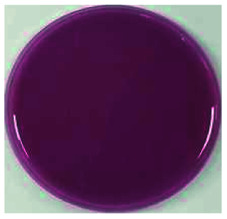	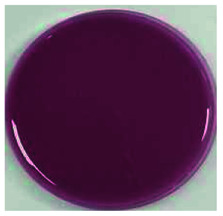

### 3.3 The total phenolics and anthocyanin contents

**Fig. 2:**
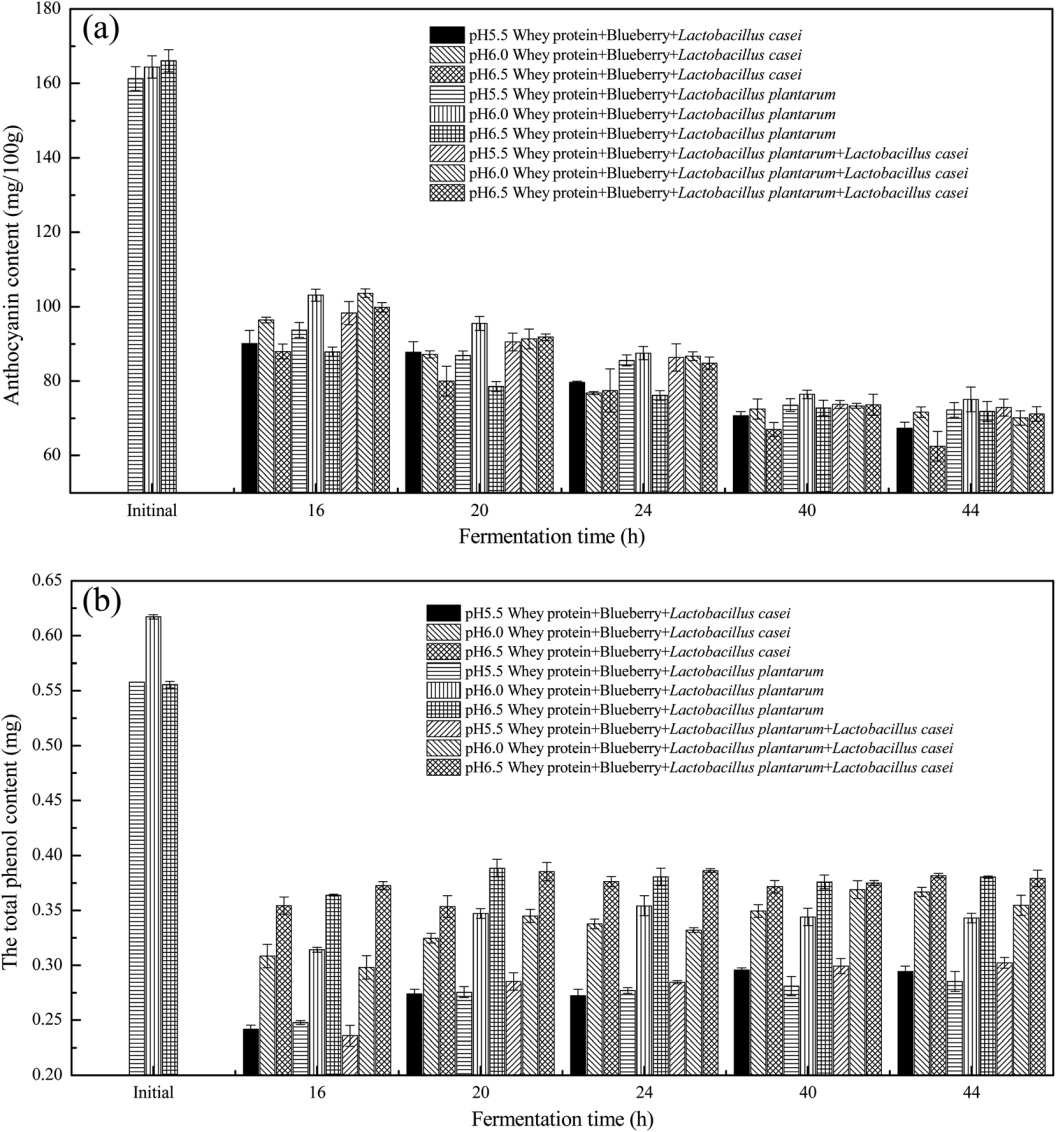
The anthocyanins content (a) and total phenol content (b) of whey protein and blueberry juice fermented using *Lactobacillus plantarum*, *Lactobacillus casei* or their combination systems with initial pH 5.5, pH 6.0 and pH 6.5 conditions at the fermentation time of 16 h, 20 h, 24 h, 40 h and 44 h.

Anthocyanins have low stability and are susceptible to environmental oxygen, heat, pH, light, organic acids, enzymes, auxiliary colorants, food additives, metal ions and sugars and their degradation products, which lead to molecular isomerization, polymerization and degradation directly affecting natural anthocyanin degradation and discoloration.^20^ As shown in Fig. 2(a), due to the high temperature of fermentation, the anthocyanins decreased rapidly in the first 16 h of fermentation until it flattened. The anthocyanin content decreased until the fermentation time was 40 h. The anthocyanin content was stable during 40–44 h. The *Lactobacillus plantarum* fermented whey protein and blueberry system at initial pH of 6.0 had a slightly higher anthocyanin content than the *Lactobacillus casei* fermented sample and the same for *Lactobacillus plantarum* and *Lactobacillus casei* fermented samples. As shown in Fig. 2(b), the total phenol content in the blueberry whey fermentation system decreased rapidly. This is related to phenol degradation, which is caused by maintaining temperature of 37 °C for a long time. Studies have shown that heat treatment causes a decrease in the polyphenol content. The total phenol content at an initial pH of 6.5 was always higher than that at an initial pH of 6.0 and higher than the initial pH of 5.5. However this gap was narrows over time. The phenol content of *Lactobacillus casei* fermented sample at initial pH 6.0 was slightly increased with fermentation time increased. The phenol content of the *Lactobacillus plantarum* and combined *Lactobacillus casei* fermented samples at an initial pH 6.5 was relative higher during the 0–44 h fermentation period. Such finding indicated that the pH values of 6.0 and pH 6.5 have a better protecting effect on polyphenols. This may be related to the gradual decrease in the pH of the fermentation system and the gradual conversion of bound polyphenols to free polyphenols.^21^

### 3.4 Fluorescence measurement

**Fig. 3:**
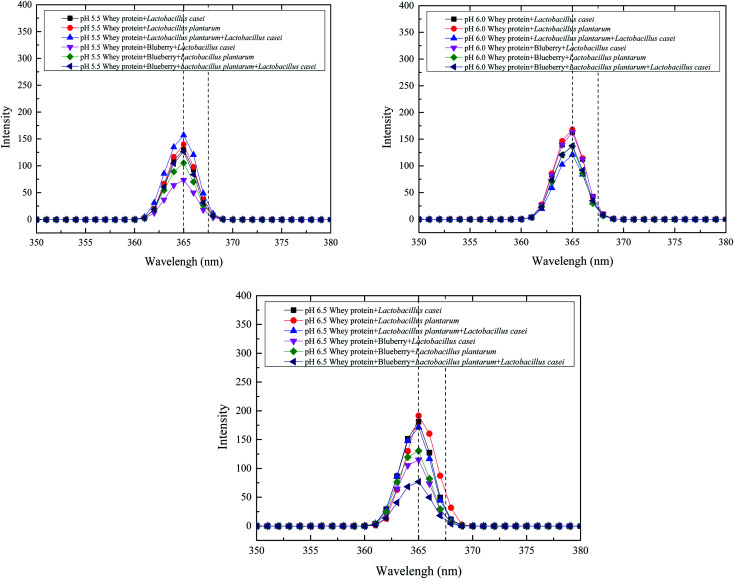
Fluorescence intensity of whey protein and blueberry juice fermented using *Lactobacillus plantarum*, *Lactobacillus casei* or their combination systems with initial pH 5.5, pH 6.0 and pH 6.5 conditions at the fermentation time of 40 h.

The tertiary structural changes induced by the application of heat treatment at different initial pH values and the levels of various monosaccharides derived from whey protein were determined using fluorescence spectroscopy. Tryptophan residues are exposed on the protein surface, as their initial maximum fluorescence emission appears to be at a wavelength close to that of exposed tryptophan model systems.^22^ The fluorescent components of whey protein are mainly phenylalanine and tryptophan. The fluorescence intensity of whey protein fermented using *Lactobacillus plantarum* was increased as the initial pH increased. The same trend was observed for *Lactobacillus casei* fermented whey protein. This finding indicated that the exposure of tryptophan of whey protein was increased with initial fermentation pH increased. However, the fluorescence intensity of whey protein fermented using *Lactobacillus plantarum* and *Lactobacillus casei* decreased at an initial pH of 6.0, which indicated that more whey protein or peptides interacted with blueberry. As shown in Fig. 3, the *Lactobacillus casei* fermented whey protein and blueberry also had a higher fluorescence intensity at an initial pH of 6.0 than 5.5 and 6.5. The same trend was observed for the *Lactobacillus plantarum* fermented whey protein and blueberry system. However, the intensity of *Lactobacillus casei* fermented system was higher than the *Lactobacillus plantarum* fermented whey protein and blueberry system at initial pH 6.0, which suggested that the addition of *Lactobacillus casei* could increases tryptophan and tyrosine exposure. Nevertheless, the combined *Lactobacillus plantarum* and *Lactobacillus casei* fermented whey protein and blueberry system had the lowest intensity at an initial pH of 6.5. One of the reasons is that the whey protein peptides interacted with the anthocyanin or phenol of blueberry reducing the fluorescence intensity. The other reason is the lower tryptophan and tyrosine exposure of whey protein. A red shift of the maximum emission wavelength was observed for *Lactobacillus plantarum*- and *Lactobacillus casei*-fermented whey protein which indicated that the spatial position of the tryptophan residues changed and the hydrophobicity of the surrounding microenvironment increased.

### 3.5 FTIR spectra analysis

**Fig. 4:**
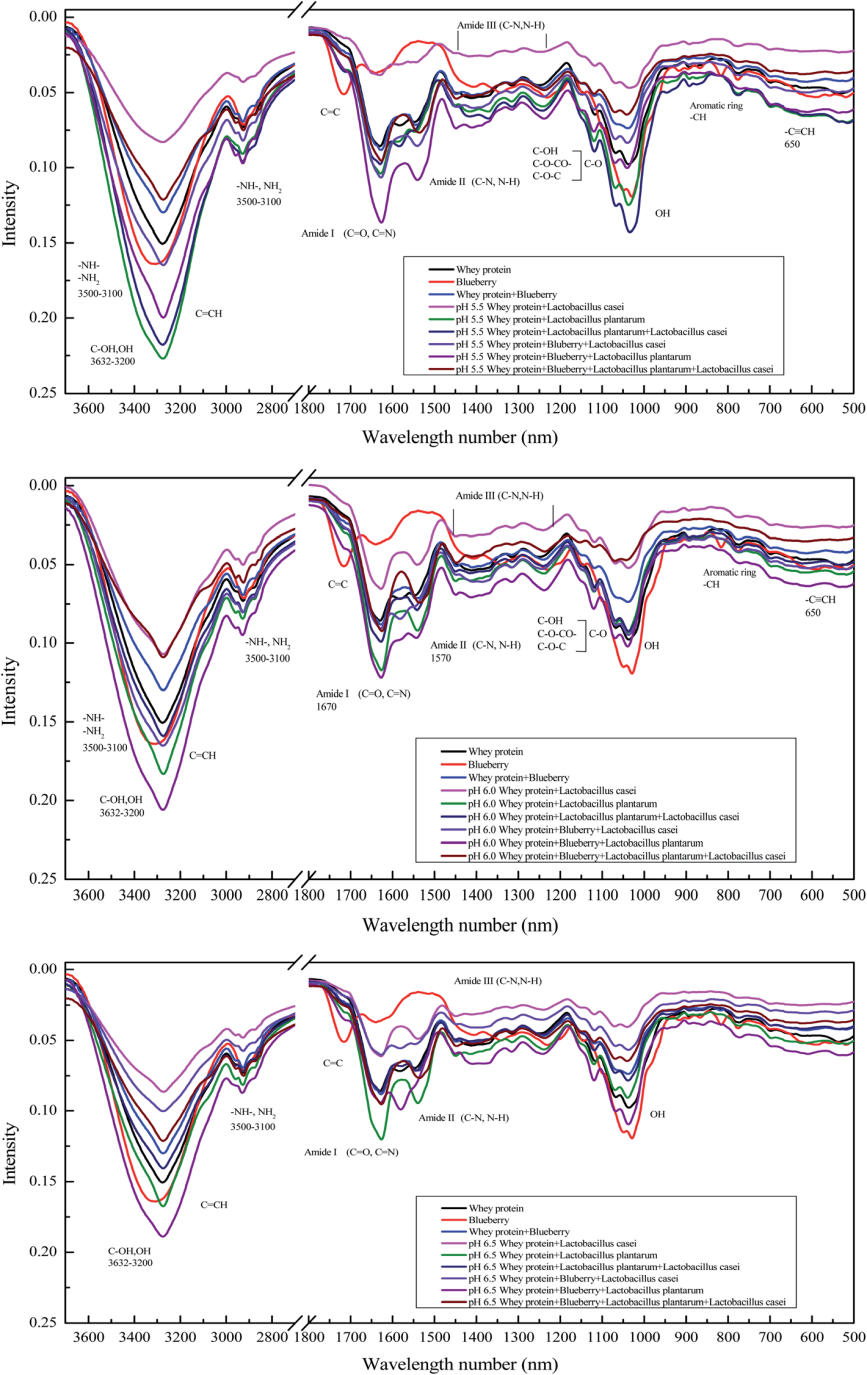
FTIR intensity of whey protein and blueberry juice fermented using *Lactobacillus plantarum*, *Lactobacillus casei* or their combination systems with initial pH 5.5, pH 6.0 and pH 6.5 conditions at the fermentation time of 40 h.

Infrared spectroscopy was used to assess the secondary structural changes occurring in the fermentation systems. As shown in Fig. 4, the amide I band (mainly C=O and C=N stretch) at 1600–1700 cm^−1^ and the amide II band (C–N stretching coupled with N–H bending modes) at 1500–1600 cm^−1^ were observed in all the samples. The spectral shifts at 1600–1700 cm^−1^ and 1500–1600 cm^−1^ indicate the secondary structural changes of whey protein.^14^ The broad peak of 1300–1200 cm^−1^ was assigned to amide III (C–N stretching and N–H deformation). The FTIR spectrum of blueberry juice shows an absorption peak at approximately about 3334 cm^−1^ (Fig. 4), assigned to the stretching and bending vibrations of the O–H groups. In addition, a strong absorption band at 1021 cm^−1^ was assigned to the aromatic ring C–H deformation. The bands at 1638 cm^−1^ and 1414 cm^−1^ corresponded to the stretching vibration of the C–C aromatic ring. The peak at 1980–1700 cm^−1^, 3000–3100 cm^−1^ and 2000–2500 cm^−1^, which correspond to C=C, C=CH and C=C=C respectively, were mainly assigned to the aromatic ring of blueberry's anthocyanins. However, the peak of 780 cm^−1^ represents the –CH stretching from the aromatic amino acid rings for whey protein. The absorption band with a maximum at 1257 cm^−1^ was assigned to the stretching of pyran rings, which is typical of flavonoid compounds. The band at 1344 cm^−1^ corresponds to the C–O angular deformations of phenols.^23^ As shown in Fig. 4, the absorption wavelength of the whey protein and blueberry combination sample slightly decreased relative to that of blueberry alone. This finding indicates an interaction between whey protein and blueberry. After fermentation of whey protein using *Lactobacillus casei*, the FTIR spectrum intensity was significantly reduced, which may be due to the hydrolysis of whey protein by microorganism. The intensity of the whey protein and blueberry fermented by *Lactobacillus casei* was slightly reduced compared to that of blueberry samples fermented using *Lactobacillus plantarum*, for which the amide I and II band intensity were slightly increased. Such finding indicated that there were more aromatic ring compounds in the *Lactobacillus casei*-fermented whey samples than in the *Lactobacillus plantarum*-fermented whey protein and blueberry samples. These aromatic ring compounds mainly contributed to whey protein. This also means that the addition of blueberry juice could inhibit whey protein degradation deeply in the *Lactobacillus casei* fermentation systems. Compared with whey protein without fermentation, the addition of blueberry juice reduced the strength of the N–H bond of proteins and that of C–O bonds in phenols, which indicates that the blueberry and the whey proteins were naturally interact with each other. The intensity of the whey protein amide group (including N–H, C=O and C–N) fermented by *Lactobacillus casei* was lower than fermented by *Lactobacillus plantarum* at initial pH values of 5.5, 6.0 and 6.5. This finding indicated that more hydroxyl groups and amino groups exposed after *Lactobacillus plantarum* fermentation. Whey protein fermented using *Lactobacillus casei* can also lead to weaker C–O bonds at an initial pH of 5.5. The peak strength of the fermented whey protein mixture (*Lactobacillus plantarum* and *Lactobacillus casei*) at 3500–3050 cm^−1^ for N–H and C–O was slightly lower than *Lactobacillus plantarum* fermented system at initial pH values of 5.5, 6.0 and 6.5. Compared with the unfermented system, at initial pH values of 5.5 and 6.0, the *Lactobacillus casei*-fermented blueberry and whey protein mixed system showed increased peak values (1700–1500 cm^−1^ and 1300–1000 cm^−1^, 3400–3000 cm^−1^ and 3000–2800 cm^−1^ (–NH–, NH_2_), respectively). Such finding indicates that the whey protein and blueberry are connected with each other by N–C or C=N after *Lactobacillus plantarum* or *Lactobacillus casei* fermentation. Overall, the strain and initial pH are the main factors that affect the group change during the fermentation process, which leads to change in amide and C–O in the system.

### 3.6 CD

**Fig. 5:**
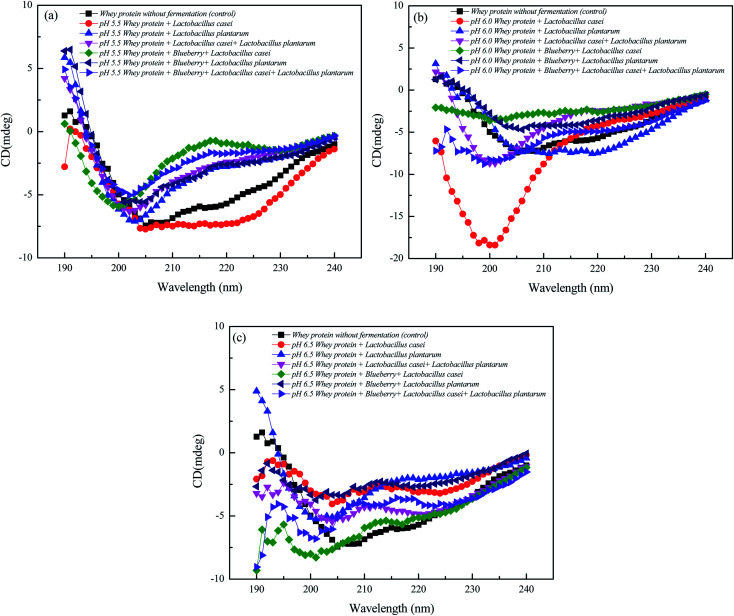
Comparative analysis of circular dichroism plots of whey protein and blueberry juice fermented by *Lactobacillus plantarum*, *Lactobacillus casei* or their combination systems with initial pH 5.5, pH 6.0 and pH 6.5 conditions at the fermentation time of 40 h.

A typical feature of a CD spectrum for a protein with an α-helices is the presence of peak at 192 nm, and the peak at 195 nm or the band at 215 nm is the characteristic of β-sheet clearly observed from Fig. 5.^16^ As shown in the Table 2, the addition of lactic acid bacteria (LAB) will change the secondary structure of whey, increasing the number of α-helices and β-sheets, while decreasing number of β-turns and random coil. The addition of blueberry juice and the differences in the strains did not significantly affect the secondary structure of whey. The whey protein fermented using *Lactobacillus plantarum* and *Lactobacillus casei* had a higher α-helix content. However, the β-sheet content of the whey protein and blueberry fermented using *Lactobacillus plantarum* did not changed significantly. However, the α-helix content decreased by 18% compared to that of whey protein fermented using *Lactobacillus plantarum* at an initial pH of 5.5. Furthermore, the β-turn content was decreased by 10%. These results might indicate that the polypeptide component was extended upon complexation with blueberry, and the secondary structures of whey protein were altered due to the binding of the anthocyanins or the phenolic hydroxyl groups of blueberry to the N–H, C–N and C=O groups of whey protein during *Lactobacillus plantarum* fermentation process.^14^ The structure of a protein is typically stabilized by four interactions: ionic bonding, hydrogen bonding, disulfide linkages, and dispersion forces. The secondary structure of a protein is mainly maintained by hydrogen bonds. *Lactobacillus plantarum* fermentation was suspected to disrupt the hydrogen bonds between β-sheets which resulting the decreased β-sheet content. The addition of blueberry slightly increased the β-sheet content. However, the random coil content of whey protein and blueberry fermented using *Lactobacillus casei* was not significant compared to that of only whey protein fermented alone. This finding means that the blueberry addition in whey protein did not affect the whey protein random coil content at an initial pH of 5.5. In addition, the fermentation of whey protein and blueberry using *Lactobacillus casei* and *Lactobacillus plantarum* had a slight effect on the secondary structure compared to only whey protein fermentation. Therefore, pure *Lactobacillus plantarum* fermentation had significant effect on the secondary structure of the whey protein and blueberry combination system. Furthermore, the influence of the initial pH was significant. The β-sheet content was the highest at an initial pH of 5.5, the α-helix content was the highest at an initial pH of 6.0, and the β-turn content was the highest at an initial pH of 6.5.

**Table 2:** The secondary structure changes of whey protein and blueberry juice fermented with *Lactobacillus casei* and *Lactobacillus plantarum* at various initial pH

Sample	α-helix (%)	β-sheet (%)	β-turn (%)	Random coil (%)
Whey protein (control)	11.17 ± 0.524^d^	39.67 ± 2.357^b^	10.67 ± 1.648^f^	38.53 ± 0.717^c^
pH 5.5 whey protein + *Lactobacillus casei*	18.20 ± 3.493^b^	14.80 ± 2.137^f^	24.90 ± 0.777^b^	42.20 ± 2.310^b^
pH 5.5 whey protein + *Lactobacillus plantarum*	22.00 ± 2.325^a^	11.20 ± 3.109^g^	25.80 ± 0.536^b^	41.00 ± 0.551^b^
pH 5.5 whey protein + *Lactobacillus casei* + *Lactobacillus plantarum*	16.90 ± 3.227^c^	16.90 ± 2.516^f^	23.40 ± 0.74^b^	42.90 ± 1.676^b^
pH 5.5 whey protein + blueberry + *Lactobacillus casei*	3.30 ± 2.968^f^	41.10 ± 2.707^a^	12.90 ± 0.667^e^	42.70 ± 2.003^b^
pH 5.5 whey protein + blueberry + *Lactobacillus plantarum*	4.00 ± 2.186^f^	38.40 ± 4.683^b^	14.50 ± 3.769^d^	43.10 ± 1.299^b^
pH 5.5 whey protein + blueberry + *Lactobacillus casei* + *Lactobacillus plantarum*	3.70 ± 2.627^f^	42 ± 3.150^a^	11.40 ± 1.391^f^	42.90 ± 1.278^b^
pH 6.0 whey protein + *Lactobacillus casei*	18.9 ± 1.841^b^	12.5 ± 0.326^g^	25.4 ± 3.006^b^	43.2 ± 3.362^b^
pH 6.0 whey protein + *Lactobacillus plantarum*	19.9 ± 1.067^b^	17.6 ± 2.058^f^	23.6 ± 4.203^b^	39.0 ± 0.633^c^
pH 6.0 whey protein + *Lactobacillus casei* + *Lactobacillus plantarum*	23.8 ± 2.146^a^	2.9 ± 1.182^h^	31.2 ± 3.486^a^	42.1 ± 2.896^b^
pH 6.0 whey protein + blueberry + *Lactobacillus casei*	11.7 ± 4.341^d^	22.2 ± 2.117^e^	20.1 ± 3.326^c^	46.0 ± 2.685^a^
pH 6.0 whey protein + blueberry + *Lactobacillus plantarum*	11.5 ± 2.149^d^	29.4 ± 0.984^d^	16.3 ± 0.746^d^	42.8 ± 3.486^b^
pH 6.0 whey protein + blueberry + *Lactobacillus casei* + *Lactobacillus plantarum*	11.1 ± 0.656^d^	28.5 ± 2.463^d^	17.2 ± 2.618^c^	43.3 ± 3.134^b^
pH 6.5 whey protein + *Lactobacillus casei*	13.6 ± 1.840^c^	21.6 ± 4.295^e^	22.3 ± 2.675^c^	42.4 ± 4.213^b^
pH 6.5 whey protein + *Lactobacillus plantarum*	23.9 ± 4.341^a^	3.5 ± 0.717^h^	27.5 ± 3.587^b^	44.9 ± 5.036^b^
pH 6.5 whey protein + *Lactobacillus casei* + *Lactobacillus plantarum*	15.2 ± 0.949^c^	22.1 ± 1.582^e^	22.1 ± 4.379^c^	40.6 ± 1.223^b^
pH 6.5 whey protein + blueberry + *Lactobacillus casei*	6.3 ± 1.077^e^	35.4 ± 1.271^c^	15.7 ± 1.269^d^	42.5 ± 3.698^b^
pH 6.5 whey protein + blueberry + *Lactobacillus plantarum*	9.8 ± 1.297^d^	28.5 ± 2.200^d^	18.4 ± 2.662^c^	43.4 ± 2.569^b^
pH 6.5 whey protein + blueberry + *Lactobacillus casei* + *Lactobacillus plantarum*	14.8 ± 1.548^c^	16.1 ± 2.277^f^	24.3 ± 3.028^b^	44.8 ± 2.336^b^

### 3.7 Microstructure

**Fig. 6:**
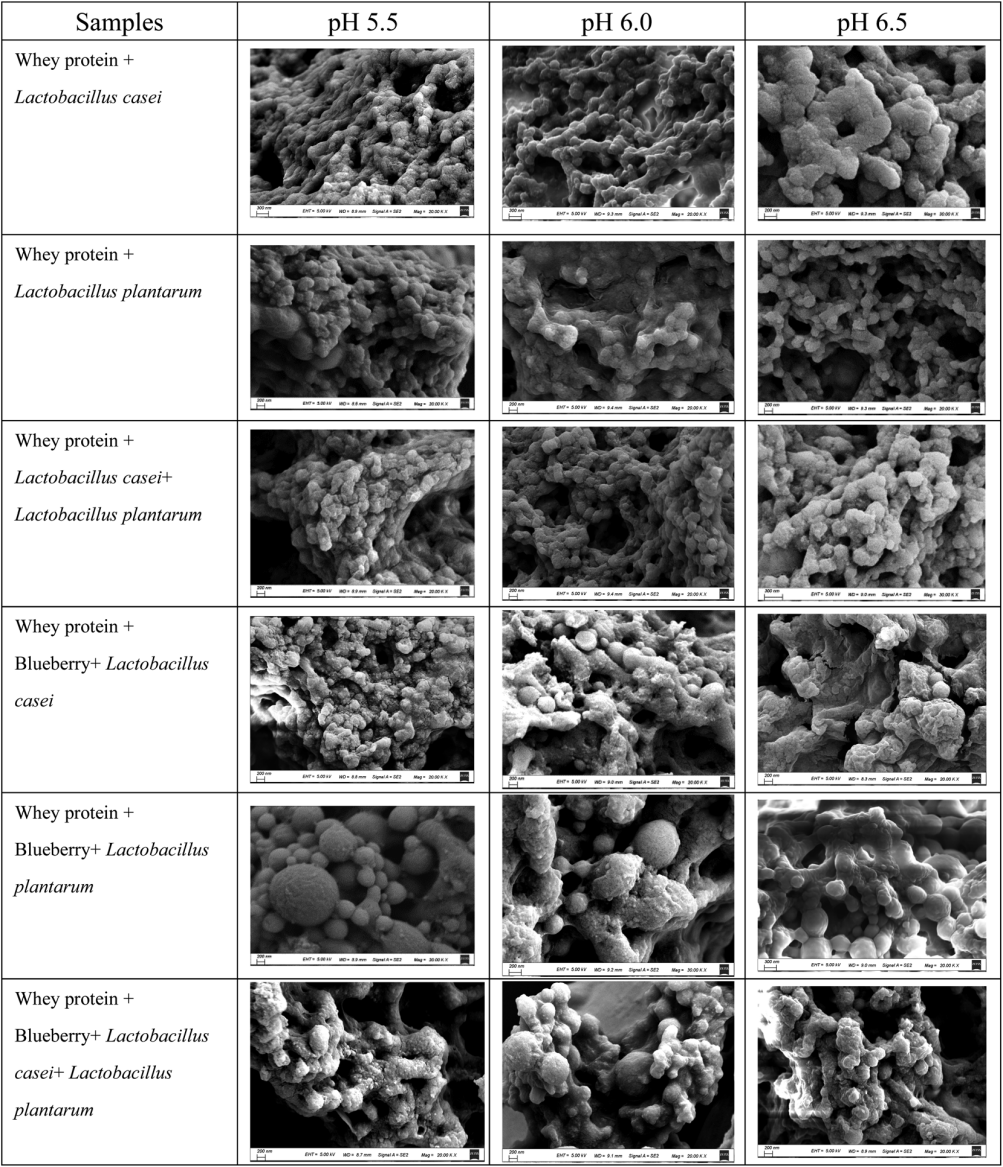
Scanning Electron Microscopy (SEM) photographs of whey protein and blueberry juice fermented using *Lactobacillus plantarum*, *Lactobacillus casei* or their combination systems with initial pH 5.5, pH 6.0 and pH 6.5 conditions at the fermentation time of 40.

The SEM image in Fig. 6 shows the surface characteristic of whey protein and blueberry fermented using *Lactobacillus casei* and *Lactobacillus plantarum*. The denatured whey protein aggregates to form a three-dimensional network at various initial pH values after fermentation. For the whey protein fermented using *Lactobacillus casei* and *Lactobacillus plantarum* separately or in combination, the network was composed of spherical structures of uniform particle size at different initial pH values, excepted at pH of 6.5 when fermented using *Lactobacillus casei*. At an initial pH of 6.5, the surface of fermented whey protein was rough and the protein cluster was larger and irregular compared to that at a pH of 5.5 or 6.0. The surface spherical structure of whey protein showed edge sharpness after fermentation using *Lactobacillus casei* and *Lactobacillus plantarum*, compared to the single strain fermentation. However, the structure of the whey protein and blueberry mixture fermented by *Lactobacillus casei* and *Lactobacillus plantarum* was irregular, with an uneven particle size distribution and some small pieces on the surface. Some large clusters were observed. This may be due to the interaction between whey protein and blueberry through hydrogen bonding or the disulfide bond between proteins.

## 4. Conclusion

Compared to the initial system, the total anthocyanin and phenol contents of the whey protein and blueberry fermentation system were decreased. However, the anthocyanin and phenol contents were stable when fermentation was carried out using *Lactobacillus plantarum* or *Lactobacillus casei* alone or in combination. The *Lactobacillus casei*-fermented whey protein and blueberry system had a higher tryptophan and tyrosine exposure. The FTIR intensity results showed that the characteristic peaks of whey protein and blueberry changed significantly using *Lactobacillus plantarum* and *Lactobacillus casei* fermentation. Compared with the *Lactobacillus casei* and *Lactobacillus plantarum* fermentation of whey protein alone, when whey protein and blueberry were fermented which has slightly effects on the secondary structure of whey protein. Compared with the whey protein fermented using *Lactobacillus casei*, the α-helix content of the whey protein and blueberry fermented using *Lactobacillus plantarum* alone increased by approximately 4% and the β-sheet content decreased by 3.6% compared to whey protein fermented using *Lactobacillus casei* which was due to the binding of anthocyanins or the phenolic hydroxyl groups of blueberry to the N–H, C–N and C=O groups of whey protein. The research shows that the whey protein secondary structure changed was related to the surface bonds and interaction with the environmental material during fermentation. A single amino acid change could have an effect on the change between a β-sheet-rich conformation and an α-helix-rich conformation.^24^

## Author contributions

Wang Wen-qiong: investigation; formal analysis; writing – original draft; Zhang Jie-long: methodology, resources of original materials; Yu Qian: CD measurement analysis; Zhou Ji-yang: FTIR measurement analysis; Lu Mao-lin & Gu Rui-xia: supervision; Huang Yu-jun: funding support.

## Conflicts of interest

The authors declare no conflict of interest.

## Supplementary Material
